# Supplementary figures and images for: Value judgment of new medical treatments: Societal and patient perspectives to inform priority setting in The Netherlands
Source: PLoS One. 2020 Jul 9;15(7):e0235666. doi: 10.1371/journal.pone.0235666 (PMC7347112; doi:10.1371/journal.pone.0235666)

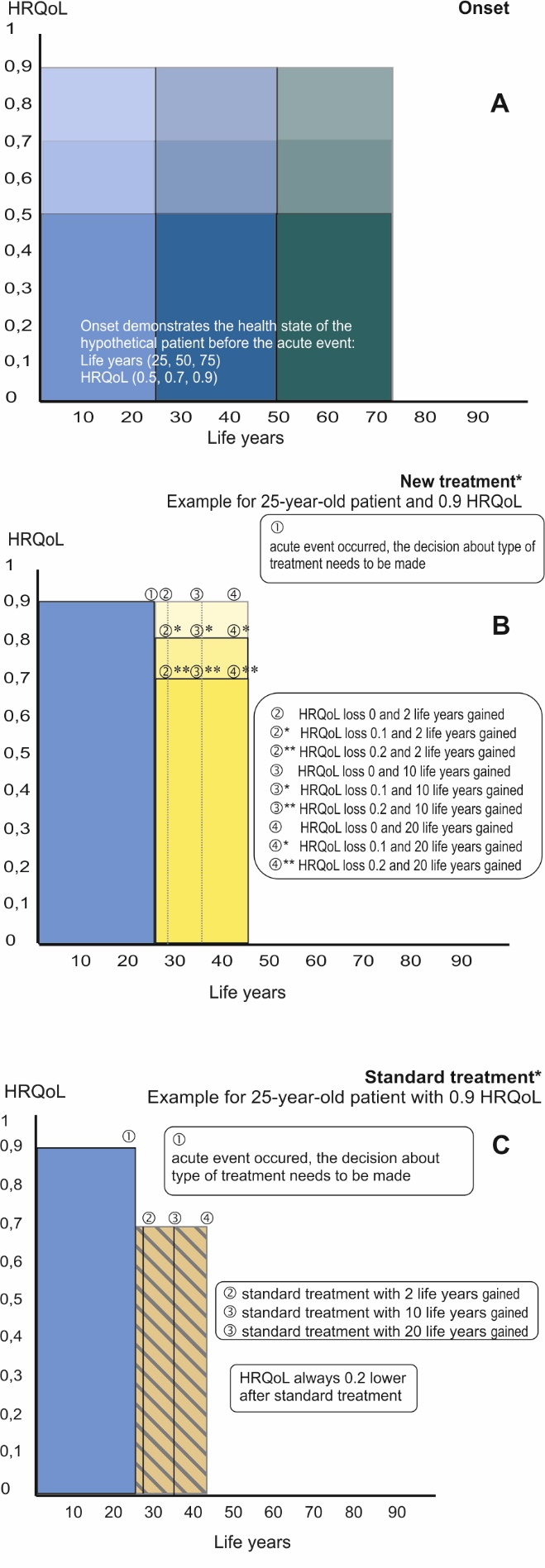

Supplement: S1 Fig — Explanation of various options in the scenarios: (A) Possible health states before onset; (B) Example of new treatment for 25-year-old patient with 0.9 HRQoL; (C) Standard treatment for 25-year-old patient with 0.9 HRQoL (if accessible and exist). (TIF) [file pone.0235666.s001.tif]
