# Supplementary material for: Value judgment of new medical treatments: Societal and patient perspectives to inform priority setting in The Netherlands
Source: PLoS One. 2020 Jul 9;15(7):e0235666. doi: 10.1371/journal.pone.0235666 (PMC7347112; doi:10.1371/journal.pone.0235666)
Supplement: S1 Table — (DOCX) [file pone.0235666.s002.docx]

**S2 Table**

Parameter estimates (mixed logit) of the 6 criteria for the two sub-samples (based on completed surveys)

|  | General population (SE)  N=1,253  Obs=47,756 | Patients (SE)  N=1,389  Obs=51,932 |
| --- | --- | --- |
|  |  |  |
| **SCENARIO CRITERIA** |  |  |
| **Patient characteristics** |  |  |
| *Age* |  |  |
| Age 25 (reference) | - | - |
| Age 50 | -0.22 (0.03)* | -0.11 (0.03)* |
| Age 75 | -0.94 (0.04)* | -0.76 (0.04)* |
|  |  |  |
| *Initial Health-Related Quality of Life (HRQoL)* |  |  |
| HRQoL 0.5 (reference) | - | - |
| HRQoL 0.7 | 0.35 (0.04)* | 0.30 (0.03)* |
| HRQoL 0.9 | 0.59 (0.04)* | 0.36 (0.03)* |
|  |  |  |
| *Cause of acute onset* |  |  |
| Accident, genetics (reference) | - | - |
| Unhealthy lifestyle | -0.89 (0.04)* | -0.75 (0.04)* |
|  |  |  |
| **New treatment outcomes** |  |  |
| *HRQoL change after new treatment (*ΔHRQoL) |  |  |
| ΔHRQoL -0.2 (reference) | - | - |
| ΔHRQoL -0.1 | 0.33 (0.03)* | 0.08 (0.03)* |
| ΔHRQoL 0 | 0.2 (0.04)* | 0.21 (0.03)* |
|  |  |  |
|  |  |  |
| *Life years gained after new treatment (LY_new_)* |  |  |
| LY_new_ 2(reference) | - | - |
| LY_new_ 10 | 0.84 (0.04)* | 0.74 (0.04)* |
| LY_new_ 20 | 1.26(0.04)* | 1.14 (0.04)* |
|  |  |  |
| **Standard treatment outcomes***** |  |  |
| *Life years gained after standard treatment (LY_standard_)* |  |  |
| Standard treatment unavailable (reference) | - | - |
| LY_standard_ 2 | -0.07 (0.03)** | -0.01 (0.03) |
| LY_standard_ 10 | -0.05 (0.04) | 0.01 (0.04) |
| LY_standard_ 20 | -0.157 (0.05)* | 0.01 (0.05) |
|  |  |  |
|  |  |  |
| Goodness-of-fit | -13442 | -15232 |
|  |  |  |

*P<0.01, **P<0.05

**** Change in HRQoL after standard treatment has no variance as it is a fixed attribute with one possible level of -0.2 (7^th^ criterion)*
