# Supplementary material for: Value judgment of new medical treatments: Societal and patient perspectives to inform priority setting in The Netherlands
Source: PLoS One. 2020 Jul 9;15(7):e0235666. doi: 10.1371/journal.pone.0235666 (PMC7347112; doi:10.1371/journal.pone.0235666)
Supplement: S2 Table — (DOCX) [file pone.0235666.s003.docx]

**S3 Table**

Parameter estimates of the 6 criteria with interaction terms (sample type × criteria)
for the combined sample of general population and patients.

|  | | General population and patients, (SE),  N=2, 642, Obs=99,834 |  |
| --- | --- | --- | --- |
|  | |  |  |
| **SCENARIO CRITERIA** | |  |  |
| **Patient characteristics** | |  |  |
| *Age* | |  |  |
| Age 25 (reference) | | - |  |
| Age 50 | | -0.17 (0.02)* |  |
| Age 75 | | -0.67 (0.02)* |  |
|  | |  |  |
| *Health-Related Quality of Life before onset (HRQoL)* |  |  |  |
| HRQoL 0.5 (reference) | | - |  |
| HRQoL 0.7 | | 0.28 (0.03)* |  |
| HRQoL 0.9 | | 0.42 (0.02)* |  |
|  | |  |  |
| *Cause of acute onset* | |  |  |
| Accident, genetics (reference) | | - |  |
| Unhealthy lifestyle | | -0.65 (0.02)* |  |
|  | |  |  |
| **New treatment characteristics** | |  |  |
| *HRQoL change after new treatment (*ΔHRQoL) | |  |  |
| ΔHRQoL -0.2 (reference) | | - |  |
| ΔHRQoL -0.1 | | 0.16 (0.03)* |  |
| ΔHRQoL 0 | | 0.25 (0.02)* |  |
|  | |  |  |
| *Life years gained after new treatment (LY_new_)* | |  |  |
| LY_new_ 2(reference) | | - |  |
| LY_new_ 10 | | 0.64 (0.03)* |  |
| LY_new_ 20 | | 0.94 (0.03)* |  |
|  | |  |  |
| **Standard treatment characteristics***** | |  |  |
| *Life years gained after standard treatment (LY_standard_)* |  |  |  |
| Standard treatment unavailable (reference) | | - |  |
| LY_standard_ 2 | | -0.04 (0.02) |  |
| LY_standard_ 10 | | -0.04 (0.03) |  |
| LY_standard_ 20 | | -0.10 (0.04)* |  |
|  | |  |  |
| **RESPONDENT TYPE** | |  |  |
| General population (reference) | | - |  |
| Patient | | 0.01 (0.01) |  |
|  | |  |  |
| **INTERACTIONS BETWEEN SAMPLE AND CRITERIA** | |  | |
| Patient × Age 50 | | 0.08 (0.03)** |  |
| Patient × Age 75 | | 0.15 (0.03)* |  |
| Patient × HRQoL 0.7 | | -0.04 (0.04) |  |
| Patient × HRQoL 0.9 | | -0.14 (0.03)* |  |
| Patient × ΔHRQoL -0.1 | | -0.12 (0.04)* |  |
| Patient × ΔHRQoL 0 | | -0.07 (0.03)** |  |
| Patient × LY_new_ 10 | | -0.09 (0.04)** |  |
| Patient × LY_new_ 20 | | -0.10 (0.04)* |  |
| Patient × LY_standard_ 2 | | 0.04 (0.03) |  |
| Patient × LY_standard_10 | | 0.05 (0.03) |  |
| Patient × LY_standard_ 20 | | 0.12 (0.05) |  |
| Patient × Unhealthy lifestyle | | 0.10 (0.03)* |  |
|  | |  |  |
| Wald chi2(25) | | 5657.42 |  |
| Prob(chi2) | | 0.0000 |  |
|  | |  |  |

*P<0.01, **P<0.05

**** Change in HRQoL after standard treatment has no variance as it is a fixed attribute with one possible level of -0.2 (7^th^ criterion)*

We tested for statistical significance of the interactions of the criteria with the respondent type (1=patient, 0=general population). The principal information of the analysis and presented in Appendix C is the contribution of the interactions. However, mathematically, the numerical meaning of the effect size of the interaction terms equals the difference between the effect size of the criteria of the general population (Appendix B) and the criteria of the patients (Appendix B). To illustrate: Unhealthy lifestyle coefficient among general population (Appendix B) is -0.65; Unhealthy lifestyle coefficient among Patients (Appendix B) is -0.55. The coefficient of the interaction term Unhealthy lifestyle Χ patient type (Appendix C) is 0.10, resulting into -0.65 + 0.10 = -0.55. Therefore, the coefficients in Appendix B and C are identical.
