# Supplementary material for: Value judgment of new medical treatments: Societal and patient perspectives to inform priority setting in The Netherlands
Source: PLoS One. 2020 Jul 9;15(7):e0235666. doi: 10.1371/journal.pone.0235666 (PMC7347112; doi:10.1371/journal.pone.0235666)
Supplement: S1 Appendix — (DOCX) [file pone.0235666.s004.docx]

**S1 Appendix.** Rationale and assumptions for selected levels

*Initial health state*

Initial health state (the state prior to the acute onset) was captured by age at onset and initial HRQoL. Age at onset was categorized as 25, 50, or 75 years given the following assumptions: age 25 is considered a relatively healthy time of life, associated with optimal level of abilities, intelligence and productivity [28]; age 50 is when illnesses or symptoms first arise and health graduately starts to reduce [29]; age 75 presents the fastest growing section of populations in the developed world, and comprises those with substantial health problems [30]. Initial HRQoL was categorized as 0.5, 0.7, and 0.9 on a scale from 0.0 to 1.0, where 0.0 stands for the worst health and 1.0 for perfect health (explained in introduction of survey). The cut-off points were based on practical considerations. Perfect health seems nearly impossible; in practice, 0.9 is as close to full health as one gets. An HRQoL of 0.7 is assumed to be reflective of an individual experiencing some health problems that limit normal functioning, such as moderate angina [31]. Poor health is indicated by 0.5, which is seen among severely ill patients [31].

*New and standard treatment*

We assumed that HRQoL after any treatment could not exceed HRQoL before the acute onset [32], so patients would not be better than before. In the most optimistic case, they would return to the health state they had before the acute disease/injury – which is likely what a treatment is aimed at in clinical practice. Thus, a change in HRQoL after the new treatment could be zero (maintained at the previous level), decrease slightly (-0.1), or decrease substantially (-0.2). For the HRQoL change after standard treatment, we assumed only a substantial decrease (-0.2). With imposing this assumption, we let the new treatment be equal to or better than the standard treatment, by definition.

The gain in life years was categorized as 2, 10, and 20 additional years after undergoing a new treatment. These gains also applied to the standard treatment. To account for situations in which a standard treatment is not available, the gain in life years for standard treatment was set to zero. The gains of 2, 10 and 20 life years were chosen to be evenly distributed across a plausible range of plausible combinations with maximum age, as Skedgel et al. [33] suggested. A minimum gain of 2 years was assumed to avoid comparisons with immediate death. In addition, a median survival of 2 years is representative for a number of malignant diseases. Ten years of life gain was considered realistic for taking a number of chronic health states in mind. A maximum gain of 20 years was assumed to yield realistic scenarios when combined with a maximum age of 75. The scenarios were constructed such that a gain in life years after standard treatment could not exceed the gain after the new treatment. This implies, as with gain in HRQoL, that the new treatment will always be equal to or better than the standard treatment, by definition.
