# Supplementary material for: Value judgment of new medical treatments: Societal and patient perspectives to inform priority setting in The Netherlands
Source: PLoS One. 2020 Jul 9;15(7):e0235666. doi: 10.1371/journal.pone.0235666 (PMC7347112; doi:10.1371/journal.pone.0235666)

Appendix 2

Example of paired scenarios in 5 choice tasks of one block (in written and graphical format)

Choice task 1

	Scenario 1	Scenario 2
**Age**	50	75
**Initial HRQoL**	0.7	0.9
**HRQoL change after new treatment**	-0.1	0
**Life years gained after new treatment**	20	2
**Life years gained after standard treatment**	2	2
**Cause**	Accident/genetics	Unhealthy lifestyle

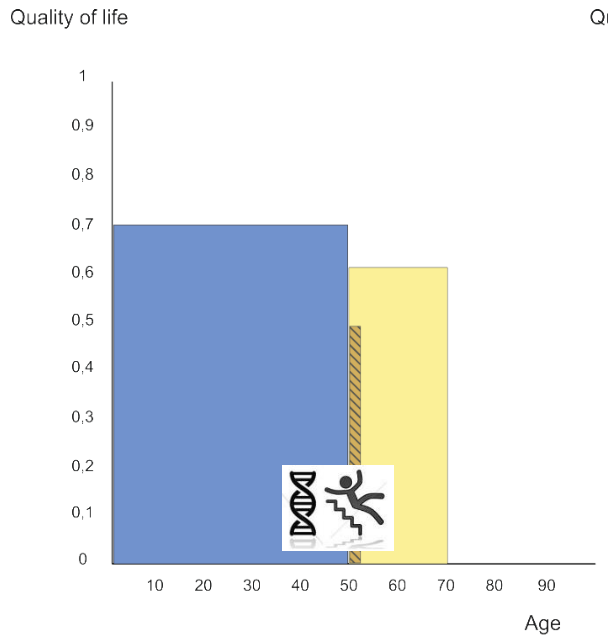

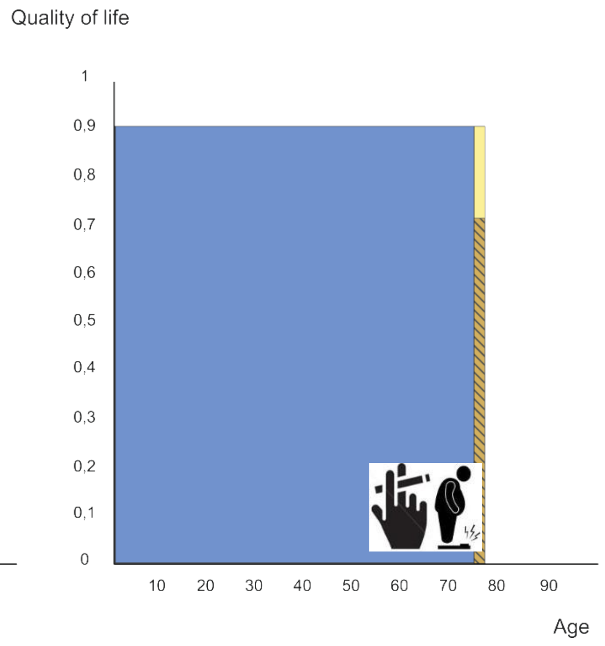

Choice task 2

	Scenario 1	Scenario 2
**Age**	75	25
**Initial HRQoL**	0.5	0.7
**HRQoL change after new treatment**	-0.2	-0.1
**Life years gained after new treatment**	20	2
**Life years gained after standard treatment**	10	0
**Cause**	Accident/genetics	Unhealthy lifestyle

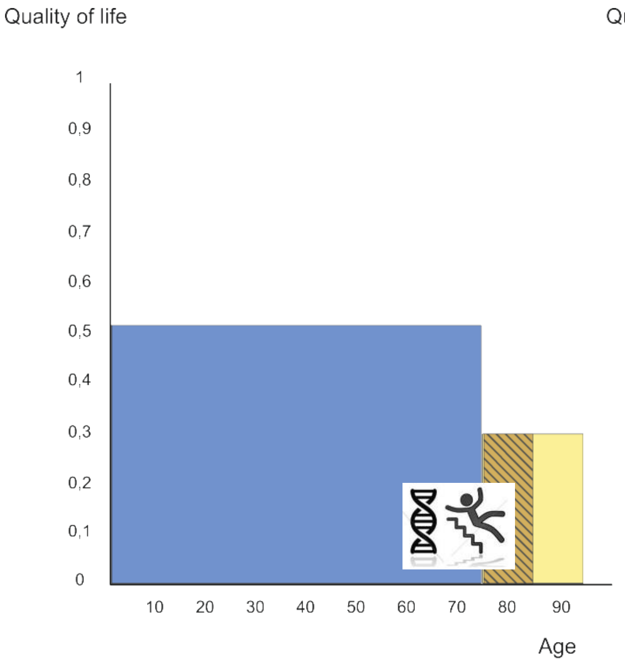

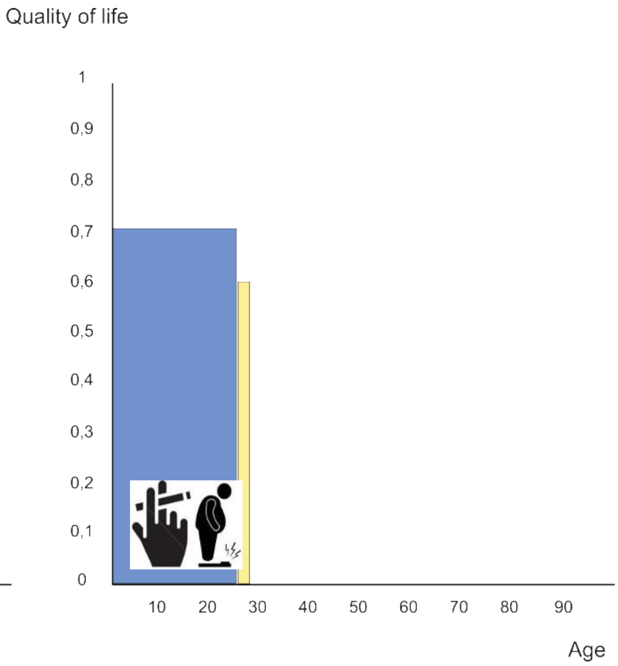

Choice task 3

	Scenario 1	Scenario 2
**Age**	25	25
**Initial HRQoL**	0.9	0.7
**HRQoL change after new treatment**	0	-0.1
**Life years gained after new treatment**	10	10
**Life years gained after standard treatment**	10	0
**Cause**	Accident/genetics	Accident/genetics

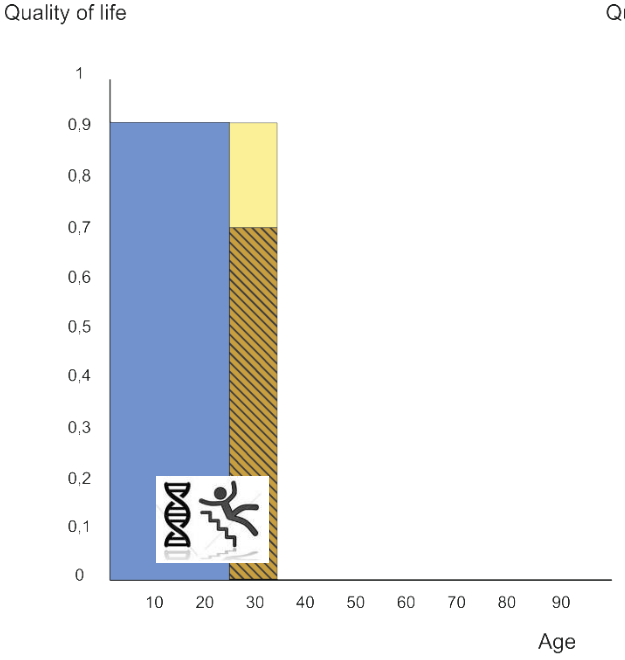

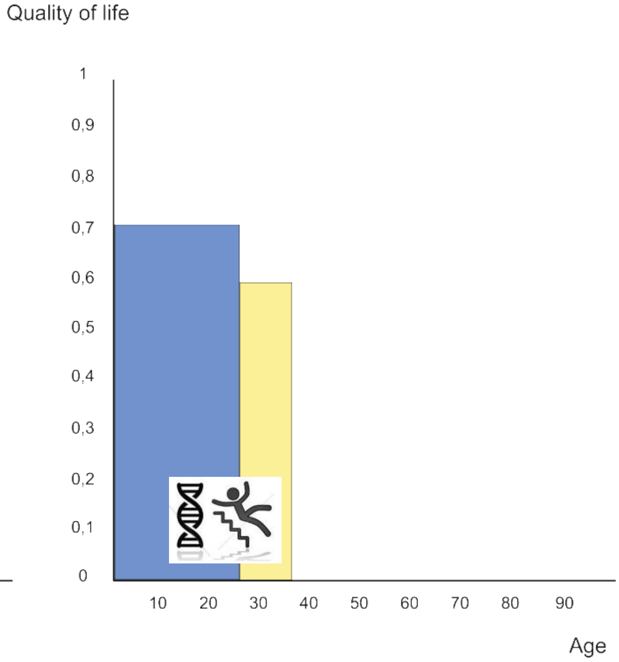

Choice task 4

	Scenario 1	Scenario 2
**Age**	75	50
**Initial HRQoL**	0.7	0.5
**HRQoL change after new treatment**	-0.1	0
**Life years gained after new treatment**	10	2
**Life years gained after standard treatment**	10	2
**Cause**	Unhealthy lifestyle	Accident/genetics

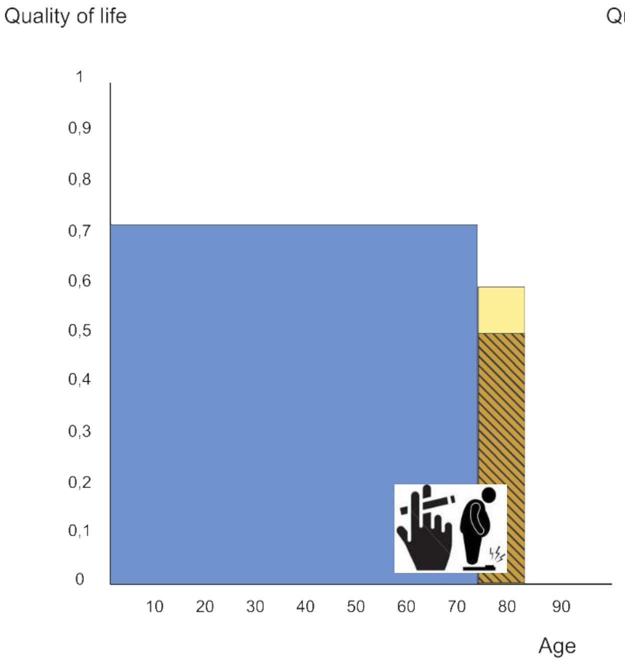

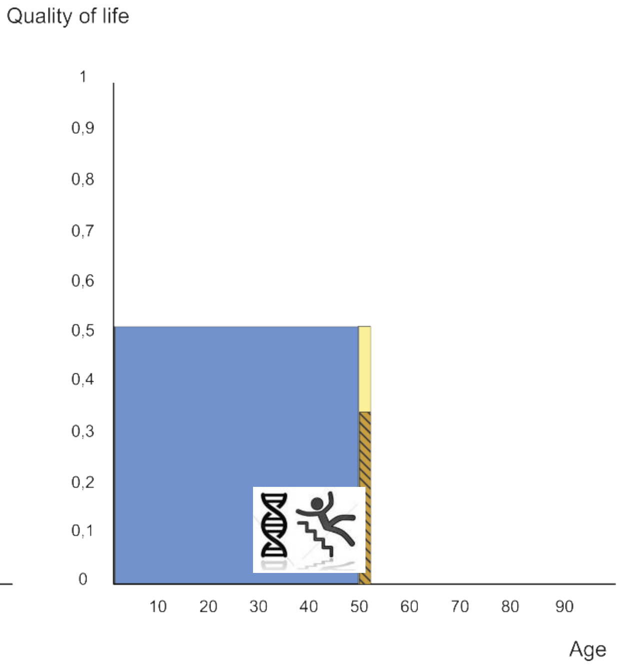

Choice task 5

	Scenario 1	Scenario 2
**Age**	75	75
**Initial HRQoL**	0.5	0.9
**HRQoL change after new treatment**	0	-0.1
**Life years gained after new treatment**	10	2
**Life years gained after standard treatment**	10	2
**Cause**	Unhealthy lifestyle	Accident/genetics

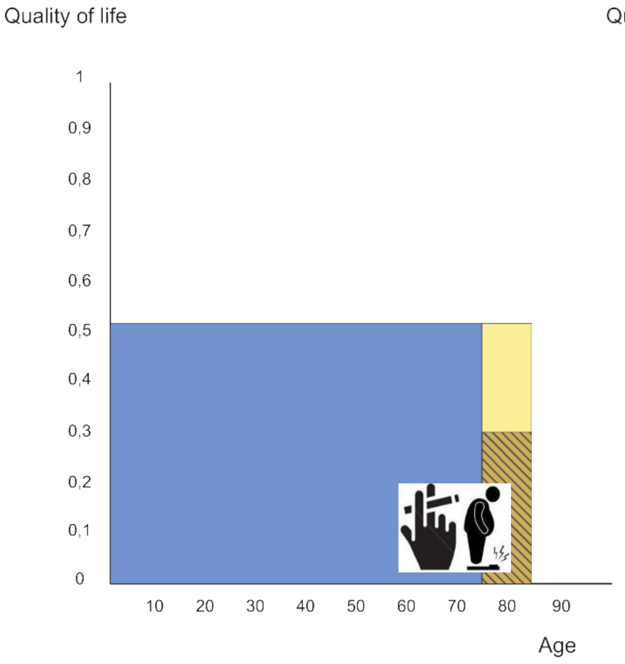

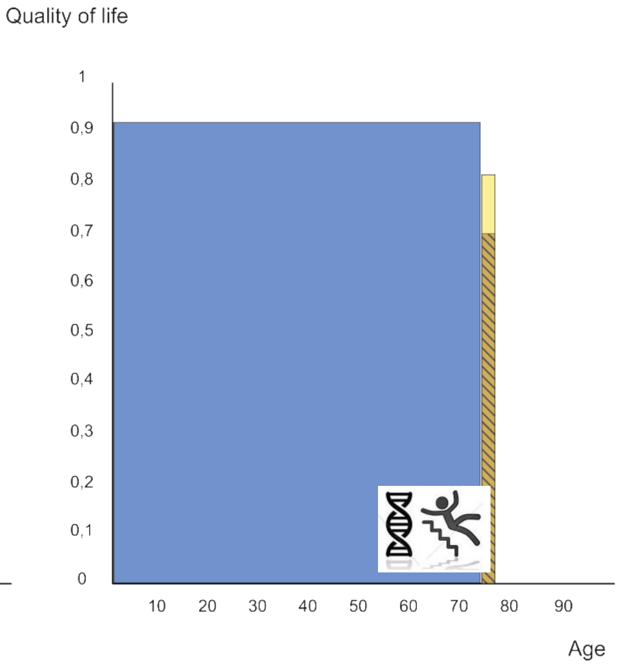

Supplement: S2 Appendix — (PDF) [file pone.0235666.s005.pdf]
