# Supplementary material for: Value judgment of new medical treatments: Societal and patient perspectives to inform priority setting in The Netherlands
Source: PLoS One. 2020 Jul 9;15(7):e0235666. doi: 10.1371/journal.pone.0235666 (PMC7347112; doi:10.1371/journal.pone.0235666)
Supplement: Attachment — Submitted filename: 46414b_corr_Response letter to Reviewer 3.docx [file pone.0235666.s006.docx]

Responses to the comments of Reviewer 3

1) How was this new approach developed? What theoretical and practical considerations and trade-offs were confronted in order to settle on the final design?

Thank you very much for raising this important issue that needs to be clarified in our paper. After reviewing the literature on priority setting, we realized that in many studies, respondents were asked to make complex choices based on scenarios presented in a verbal format, which could prove demanding for them. Therefore, we decided to develop a study design that would present different health outcomes relevant to the context of the Netherlands in a manner that was innovative and easy to understand. We conducted a comprehensive literature review to determine the optimal number of criteria that could be depicted on a graph and processed by respondents within one scenario for health outcomes. The criteria were assessed by each of the authors individually, and a group discussion was held to develop a final set of criteria. The following text has been added to the manuscript on p. 5 lines 137-151:

*“In light of our review of the available studies, we decided to present the relevant health outcomes in a way that enabled them to be easily understood. The conventional QALY concept served as a starting point for developing this approach. We created a graphical representation of a QALY (including levels and durations of particular health states) that was extended to incorporate other relevant and important criteria. To ensure that our selection of criteria (attributes in the DCE) was appropriate and well-informed, we performed an extensive review of the literature (publications from 2014 onward) using the Medline and Embase databases. The review was conducted with the aim of extracting a set of criteria that reflected societal concerns about treatment effectiveness and equity considerations [26].*

*Presenting all of the criteria (n = 25) within a single choice task would have made the task too demanding for the respondents, and many of the criteria were only applicable in very specific circumstances. Therefore, each of the 25 criteria was individually assessed by two authors and one senior researcher within the research team, and differences in their assessments were discussed to reach a consensus. Finally, a combination of essential and prominent criteria that are receiving attention within the Dutch (and international) social value appraisal system were selected.”*

2) How did participants find using the approach? Did any of them experience difficulties? Did others like the approach? The survey did not seem to ask participants what I would have thought are two obvious questions: “How easy / difficult did you find answering the choice questions?” “How confident are you in your answers?” The answers to these questions would be interesting. They might also shed some light on your results with respect to the relative importance of the attributes.

In the main survey, we did not include a field for eliciting feedback, as we assumed that changes could not be made to the survey design during this phase. Such questions and efforts to obtain feedback only occurred during the pilot phase of the study to enable us to make necessary adjustments that would improve the main survey. We added the following text to explain this process on p.7 lines 184-193:

*“Face-to-face pilot testing of the survey was performed at the University of Groningen as well as online in April 2015. There was no time limitation set for the pilot testing, although the mean duration of the face-to-face version, including instructions and feedback, was estimated to be 20 minutes. The online version was typically shorter.*

*During the pilot phase, the following questions were verbally posed to the respondents (n = 8): Were the tasks easy? Was the presentation attractive and comprehensible? Were you able to make a choice? The respondents indicated that the task was not difficult to understand, and they confirmed that the graphic design enhanced comprehension of the task. The respondents also provided some suggestions for improving the instructions provided with the task.”*

However, we agree that this issue needs to be addressed in a discussion on the study’s limitations. Therefore, we added the following text to the discussion section on p.18 lines 464-468:

*“During the pilot-testing phase, we confirmed the ease of comprehension and the appeal of our study design. However, providing the respondents with an additional opportunity to leave their feedback about the survey design and the challenges they faced could enhance the quality of the responses. These additional inputs would contribute to a deeper understanding of how the respondents executed the choice tasks and the decision strategies that they used.”*

3) Overall, is the method valid and reliable? What improvements (refinements) might be made for future uses of the method? (Should the method be used again?)

We believe that the graphical methods are appropriate for use in online DCEs, especially when respondents are required to make complex choices. Moreover, our method of gradually familiarizing respondents with the tasks and adding explanations by way of balloons and notes in the online survey proved useful at the pilot stage. However, researchers need to be aware of certain challenges, such as the differing levels of attention paid to icons and pictures versus graphs and diagrams. For example, the respondents were found to pay more attention to larger areas of the graph or to more colorful and easy-to-understand elements. Therefore, we have recommended making the following refinements to the design in the discussion section on p.18 lines 457-463:

*“Moreover, the icons could be perceived differently when presented together with a graph or a diagram. As colorful and easy-to-understand elements, icons may initially capture the attention of respondents. Consequently, respondents may pay more attention to an attribute that is presented as an icon than to the same attribute presented in a conventional textual format. Therefore, it would be useful to provide respondents with step-by-step visual guidance and verbal or even video-recorded instructions that effectively discourage them from ignoring any of the elements on the screen.”*

4) “PRESUMABLY easier to comprehend and MAY reduce certain types of bias”?! Based on just one reference? And where is reference 64?

Although there have been debates on the added benefits of a graphical format (we have added references), we opted for a more innovative graphical format compared with the conventional textual format. It has been suggested that for online-based surveys in which respondents are required to make complex choices between health-outcome scenarios, diagrams and other graphical formats are relatively easy to comprehend and would prove useful in DCEs.

The following additional references and explanations have been added to the methods sections on p.6 lines 172-179:

*“There have been numerous debates regarding the added benefits of using graphical representations to enhance task comprehension in DCEs [27-31]. Specifically, it has been suggested that diagrams and other graphical representations could facilitate respondents in making complex choices among health states in DCEs conducted online [31]. Although not all attributes can be presented graphically, the most important attributes (length of life, quality of health/HRQoL, and additional gains or losses in life years) can be depicted in this way. Therefore, instead of using conventional textual descriptions in the DCE, we opted for graphical representations of the scenarios, which were expected to enhance respondents’ comprehension.*

5) Are there any drawbacks of using graphs and icons? (It seems obvious to me that there are!) Are there any concerns about participants’ interpretations (surely, meanings and interpretations of images/icons is a notoriously tricky area)? We are not told how you settled on the designs you used … Did you test alternative presentations?

There are some concerns about participants’ understanding and perceptions of the graphical format. A graphical approach can be potentially helpful in illustrating complex choice tasks that relate to societal problems, but presenting choice tasks in this way may have contextual effects on responses and may unintentionally influence them. This point has been included in the discussion on p. 18 lines 454-457:

*“However, a graphical representation could plausibly influence the respondents to some extent. For example, the design could differentially influence the decisions of respondents who focus on the sizes of the graph areas compared with those who focus more on instructions.”*

The following text has also been added to the discussion on p.18 lines 457-461:

*“Moreover, the icons could be perceived differently when presented together with a graph or a diagram. As colorful and easy-to-understand elements, icons may initially capture the attention of respondents. Consequently, respondents could pay more attention to an attribute that is presented as an icon than to the same attribute presented in a conventional textual format.”*

6) Is it possible that requiring participants to, first, notice and understand the six criteria represented in Figure 1, and then cognitively process them and the associated trade-offs, is asking too much of most people? Might the cognitive burden be too much? Apart from the eight pilot-testers, how would you know? Will readers be able to understand the approach as you have explained it in the paper? You need to explain to your readers how your approach works, in particular, how to interpret Figure 1.
Are the images in the software dynamic? On page 7 we are told that “three steps” were involved. Maybe the approach is obvious when working on a computer screen?

Thank you very much for this observation. We do realize that the task of interpreting Fig 1 on its own is cognitively challenging. We therefore applied a three-step approach in the online survey that was designed to lead the respondents to the final response task. The first step was to depict a hypothetical patient’s health state, revealing only the initial HRQoL (Health-Related Quality of Life) and age before the acute onset. In the second step, the respondents were informed of the gains in terms of additional years and HRQoL as the outcomes of the new treatment. During the third and final step, respondents were introduced to the actual choice task. They were informed about the availability of a standard treatment and were asked to express their preferences. The first and second steps have been added to Fig 1 to improve clarity. Additionally, we added arrows and balloons to the screen image, indicating where the gains from the standard treatment or the new treatment could be found and explaining the meaning of the icon. All of these procedures were implemented in combination to decrease the burden on the respondents. However, these changes have only been implemented online, and the balloons and arrows are not all reproducible offline. The following explanatory text has been added on p.8 lines 215-228:

*“To reduce the cognitive burden on respondents, a three-step approach was implemented that facilitated their understanding of the distinct attributes in the scenarios. As they progressed through the three consecutive steps, the respondents gradually gained familiarity with each attribute before they finally encountered the actual choice task. The first step (see Fig 1a) presented a hypothetical patient’s age and initial health state, depicting this individual’s HRQoL before the occurrence of the acute event. The second step introduced new information, namely gains from the new treatment in terms of additional life years and post-treatment HRQoL (Fig 1b). Only when they reached the third step (Fig 1c) did the respondents encounter the final scenario comprising the patient’s health state before the onset of the acute event, the benefits of the new treatment, and the benefits of the standard treatment, if this information was available. … Additionally, arrows and balloons were introduced to explain where the gains from the standard treatment or the new treatment could be found on the screen.”*

7) There seems to be no ‘data quality controls’ at all – such as the time taken to answer the questions, consistency checks (repeated questions; or the inclusion of dominated ones), counts of participants clicking the same answer (left or right patient) every time. Overall, how do you know that you have ‘good quality’ data? (Does it matter?)

We decided not to include consistency checks because of the relatively large number of questions. Moreover, we used two introductory slides to lead the respondent to the actual choice task. The first depicted the situation at the onset of the acute event and showed the patient’s age and HRQoL. The second presented the benefits of the new treatment. Therefore, the overall length of the survey was relatively large. The survey was designed in such a way that after answering the series of questions, the participants were able to return to the beginning and change their responses, as they would have become more familiar with the tasks and developed their own response heuristics. However, we did include certain checks. Our analysis revealed that only 36 respondents consistently provided the same answers (they only chose option 1 or 2). Nevertheless, we decided to retain these respondents in the sample as there were no dominant scenarios and the respondents may still have made credible choices. We added the following explanations in the text on p. 11 lines 304-309:

*“We performed a quality check of the responses by identifying respondents who consistently chose either the left or right options as their responses. Only 36 respondents (0.01%) demonstrated this response pattern. Therefore, we decided to retain these respondents in the main analysis, as such a modest proportion would not bias the main results. Additionally, because the study design did not include any clearly dominated scenarios, we could not rule out any kind of pattern as being invalid.”*

Only the overall duration of the entire survey was available, which encompassed the time taken by the SSI team to ask the pre-screening questions and the duration of the actual DCE tasks. Therefore, we decided not to impose time restrictions when analyzing the responses, as they would not show the duration of completing the DCE only part. In general, a major strength of such an assessment method, as DCE, is that if respondents provide random responses (with no underlying structure), this will not affect the information relating to the estimated parameters; only the precision level for these parameters will be reduced.


**Other Comments**

1) In the second-last paragraph of page 13 you mention a surprising result: “This is not in line with the findings of our study, which revealed that the patients with higher initial HRQoL were favored over those with lower initial HRQoL.” Any ideas why?

The explanation has been revised as follows on p. 16 lines 403-412:

“*This finding does not accord with our own findings, which showed that those patients whose initial HRQoL levels were higher were favored over those whose initial HRQoL levels were lower. There are two possible explanations for this finding. The first explanation could be that the respondents were inclined to favor younger and generally healthier individuals because they are economically and demographically productive. This strategy would therefore contribute the most to overall economic well-being within society. A second possible explanation relates to the HRQoL loss of 0.2 after the acute event that was depicted in the graph and how it was visually perceived. A level of HRQoL that is initially high would still appear high, even after a loss of 0.2 or 0.1 (in a large area of the graph), compared with an initially low HRQoL, which could worsen or remain at a low level (in a smaller area of the graph).*

2) At the top of page 14, you say, “respondents did not prefer innovations targeting people with ‘drug addiction’ and ‘obesity’”. Do you mean this? Or instead do you mean, “respondents preferred that innovations did not target people with ‘drug addiction’ and ‘obesity”? (I don’t think these two statements are the same.)

It has been shown that certain sections of the population do not endorse innovations that target individuals with obesity and drug addiction issues. In other words, they are negatively inclined toward funding innovations targeting individuals with obesity and drug addiction issues. Therefore, we have revised the text to clarify this point on p. 17 lines 422-423: “…*the researchers reported that the respondents were negatively inclined toward funding innovations that targeted individuals with drug addiction and obesity issues.*

**Typos and Stylistic Errors**

*1) P. 2: “collected from general population” should be “collected from the general population”.*

Thank you. We have corrected this error on p. 2 line 55.

*P. 2: “Preferences were strongly and significantly affected by …” is too vague. Do you mean “the relative importance of the attributes …”?*

Thank you for pointing this out. The sentence has been clarified as follows on p. 2 lines 57-59: *“The respondents’ preferences regarding new medical treatments revealed that they attached the most relative importance to additional survival years, age at treatment, initial health condition, and the cause of disease.”*

*P. 2: “in the assessment of prioritizing new medical interventions” does not read well*.

This text has been removed in the revised manuscript.

*P. 3: “which consist of direct comparison of health care interventions to determine which work best for which patients and which pose” should be “which consists of direct comparison of health care interventions to determine which works best for which patients and which poses”.*

Thank you. We have revised the text as suggested on p.3 lines 77-79.

*P. 3: “value judgements into their consideration” should be “value judgements in their consideration”.*

Thank you. We have revised the text on p.5 lines 92-93.

*P. 4: “Summarizing” would be better as “In summary”.*

Thank you. We have revised the text as suggested on p. 4 line 115.

*P. 4: Add “for the Netherlands” to the end of the phrase, “Summarizing [In summary], a clear set of criteria to guide priority setting is lacking”.*

Thank you. We have made this change on p. 4 line 115.

*P. 5: “among two (paired)” is inelegant.*

Thank you. We have removed “paired” on p. 5 line 128.

*P. 10: “Of the total amount of respondents” is also inelegant. How about just, “Of the respondents”?*

Thank you for your suggestion. We have changed the text on p.11 lines 301-302 to: “*Out of all of the individuals who registered for the survey*. …”

*P. 11: Perhaps “the importance of the criteria” should be “the relative importance of the criteria”? (And elsewhere.)*

Thank you. Where applicable, we have changed the text to “*relative importance*.”

*P. 13: “gained life years” should be “life years gained”.*

Thank you. We have revised the text accordingly on p.15 lines 384-385.

*P. 14: “These finding triggers” should probably be “These findings trigger”.*

Thank you. We have changed the text on p. 17 line 433 to “*These findings trigger the interest ….*”

*P. 14: This sentence does not make sense: “In the present study we assumed that a benefit due to the relative easiness and attractiveness of the graphical format and improving it by visual aids, such as notes and balloons, popping up as additional help.”*

Thank you for pointing this out. The sentence has been changed as follows p. 18 lines 452-454*:*

*“In the present study, we assumed that a graphical format would add value because it is attractive and relatively easy to comprehend. We also assumed that the use of supplementary visual aids, such as pop-up notes and balloons, would improve the respondents’ understanding.”*
